# Obesity simulation suits in health professions education: a scoping review

**DOI:** 10.1186/s12909-026-09565-1

**Published:** 2026-06-02

**Authors:** Anna C. Steinacker, Lukas Bischof, Daniela Herchet, Victoria Kreiss, Michael Klingenberg, Stefan Bösner

**Affiliations:** 1 Department of Health Sciences, University of Applied Sciences Fulda, Leipzigerstr. 123, Fulda, 36037 Germany; 2 Department of General Practice, Philipps University Marburg, Karl-von-Frisch-Str. 4, Marburg, 35043 Germany

**Keywords:** Simulation suit, Obesity, Empathy education, Healthcare education

## Abstract

**Background:**

Weight-related stigma toward people living with obesity is widespread in healthcare settings and has been documented among students and professionals across health disciplines. Such stigma may negatively affect communication, clinical decision-making, and quality of care. Educational strategies aimed at addressing weight bias and fostering empathy during the training of health professionals are therefore of growing interest. In recent years, obesity simulation suits have been introduced as experiential learning tools intended to sensitize learners to physical and psychosocial challenges associated with higher body weight.

**Methods:**

A scoping review was conducted in accordance with the methodological framework proposed by Arksey and O’Malley and further refined by the Joanna Briggs Institute (JBI). A comprehensive search was conducted in November 2025 in CINAHL, PubMed, Embase, and Web of Science, supplemented by citation tracking, with no restrictions on publication year or language. Titles, abstracts, and full texts were screened independently by two reviewers. Studies were eligible if they reported on the educational use of obesity simulation suits, including related terms such as bariatric, empathy, or fat suits, as well as weighted vests, within healthcare-related education involving adult learners. Data were charted using a standardized extraction form and synthesized descriptively.

**Results:**

Twelve studies met the inclusion criteria out of 152 identified records. Studies were conducted across multiple countries, including the United States, Germany, and other countries, and involved learners from medicine, nursing, nutrition science, and exercise science. OSS were either worn by learners or by standardized patients. Quantitative outcomes included attitudes toward obesity, beliefs about causation, empathy, cultural humility, and implicit weight bias, assessed using validated questionnaires. Results were mixed, with some studies reporting short-term improvements in specific measures, while others found no significant changes. Qualitative findings described increased awareness of physical effort and mobility limitations, as well as emotional responses such as discomfort.

**Conclusions:**

Existing literature on obesity simulation suits in healthcare education is limited and heterogeneous. While OSS may offer opportunities for experiential learning, there is currently no consistent evidence of sustained improvements in attitudes or empathy toward people with obesity relating to OSS. Further research is needed to clarify how OSS can be integrated thoughtfully into educational programs.

**Supplementary Information:**

The online version contains supplementary material available at 10.1186/s12909-026-09565-1.

## Introduction

Stigma within healthcare is increasingly recognized as a barrier to professional patient care [1–3]. Negative assumptions about a person’s body shape, health behaviours, or perceived responsibility for their condition can influence clinical judgement, the quality of interpersonal interactions, and ultimately health outcomes [1, 4]. Among the conditions most strongly affected by such biases is obesity, which is frequently framed through moralizing narratives that emphasize personal failure rather than complex biological, social, and environmental determinants [5]. These attitudes are not restricted to the general public: evidence shows that weight-biased beliefs are also prevalent among healthcare professionals and students across disciplines, including medicine [6], nursing [7], nutrition and dietetics students [8], and other allied health professions [9, 10]. When such biases are common in clinical practice, they can result in delayed diagnoses, reduced empathy, and diminished quality of care for patients [1, 3, 4, 11].

To counteract the negative impact of weight stigma, healthcare education increasingly emphasizes the importance of understanding lived experiences of individuals with obesity. Traditional teaching approaches such as lectures, online modules, or case-based discussions offer theoretical knowledge but might not be useful in teaching empathy or challenging stereotypes [12, 13]. As a result, educators have explored more embodied and experiential approaches. These include reflective exercises, using virtual-reality (VR), interactions with standardized patients, and, in some programmes, the use of simulation suits [12].

Obesity Simulation Suits (OSS) are wearable simulation devices designed to approximate certain physical characteristics associated with higher body weight. Most suits consist of weighted components, e.g., integrated sandbags or modular weight inserts, positioned around the upper and lower body to mimic increased mass and weight distribution. In addition, they typically include an outer foam or textile layer that reproduces larger body circumference [14, 15]. When worn, OSS can create experiences of restricted mobility, reduced range of motion, increased exertion during everyday movements, and challenges related to balance and gait. While these suits cannot replicate the full physiological or psychosocial experience of living with obesity, they allow learners to gain embodied insight into selected physical aspects of higher body weight within a controlled educational setting. By temporarily simulating aspects of navigating the world in a larger body, these suits aim to provoke reflection, stimulate perspective-taking, and raise awareness of weight-related barriers encountered in daily life [16]. Their appeal in educational settings lies in their relative simplicity, low cost, and potential for immediate experiential impact [17].

However, their use is not without controversy. Critics argue that OSS may inadvertently reinforce reductive notions of obesity by emphasizing physical limitations while overlooking psychological and social dimensions, such as internalized stigma, structural discrimination, or the long-term process of bodily change [15, 18].

Against this background, existing evidence on the educational use of obesity simulation suits appears scarce, drawing on varying outcomes, diverse learner populations, and inconsistent intervention designs. To date, no comprehensive synthesis has mapped how OSS are used in healthcare education, what learning outcomes have been reported, or how researchers and educators justify or critique their use. The purpose of this scoping review is therefore to systematically examine the breadth of published evidence on OSS in healthcare education, identify key characteristics of existing interventions, summarize reported effects, and highlight areas in need of further conceptual or empirical development.

This scoping review aims to synthesize the available literature on the use of OSS in healthcare education. The following research questions guide this review:

1. Who are the target populations for OSS in healthcare education, and at what stage of training (undergraduate, postgraduate, or continuing professional development) are they employed?
2. In what ways are OSS incorporated into educational activities, and what additional teaching or learning methods (if any) are combined with their use?
3. How do learners respond to the experience of using OSS, in terms of their reported perceptions, attitudes, and learning outcomes?
4. What limitations or concerns related to the use of OSS in healthcare education are reported in the literature?

## Methodology

### Research design

This scoping review was conducted following the methodological framework proposed by Arksey and O’Malley and later refined by Peters et al.. Scoping reviews are designed to systematically map the breadth and depth of available evidence, clarify key concepts, and identify gaps in the existing literature [19, 20].

An a priori protocol has been developed and was published on Open Science Framework (OSF) (https://osf.io/hjm6w/overview). All methodological decisions outlined in this protocol guided the conduct of the review. This scoping review was reported in accordance with the Preferred Reporting Items for Systematic Reviews and Meta-Analyses extension for Scoping Reviews (PRISMA-ScR) [21]. The completed PRISMA-ScR checklist can be seen in Additional File 1.

### Information sources and search strategy

The literature search was conducted in three steps in November 2025. In a first exploratory step, relevant keywords were identified in order to get an overview of the topic and to refine the terminology for the systematic search. This was achieved through initial searches in Google Scholar and PubMed using broad terms such as variations of “obesity suit” in combination with professional groups (e.g., medical students, nursing students). Several relevant studies were identified during this stage and served as references for developing the full search strategy. In the second step, a comprehensive and systematic database search was performed. The following electronic databases were searched: CINAHL, PubMed, Embase and Web of Science. Search strategies combined controlled vocabulary (e.g., MeSH) with free-text keywords. The full search strategy can be seen in Additional File 2. No restrictions were placed on publication year or language. In this step, a librarian from the University library in Fulda was consulted to check the search strategy. In the third step, citation tracking was applied to the studies identified in the first two steps. This included both backward citation searching (manually) and forward citation searching (using Google Scholar). Grey literature was primarily identified during the exploratory search in Google Scholar and through citation tracking. No dedicated grey literature databases were searched.

### Eligibility criteria

The eligibility criteria for this review were structured according to the Population–Concept–Context (PCC) framework [20]. The population of interest were undergraduate and postgraduate students enrolled in healthcare-related programs, including medicine, nursing, nutrition sciences, physiotherapy, occupational therapy, pharmacy, dentistry, exercise science and allied health professions. Studies were eligible when learners participated in an educational activity that was designed or facilitated by educators.

The concept of interest was the educational use of an OSS, also referred to in the literature as an obesity suit, fat suit, bariatric suit or obesity empathy suit. To be eligible, the simulation suit or device had to constitute a central component of the learning activity. Studies were excluded if obesity was simulated solely through other means, such as VR applications, lecture-based interventions without a physical suit, or clinical encounters with real patients living with obesity where no simulation device was used. For the purposes of this review, interventions using weighted vests to simulate additional body mass were also considered eligible, provided they were used to replicate the physical challenges of obesity and formed an integral part of the educational activity. This review focused exclusively on simulations involving adults. Studies targeting childhood obesity were excluded, as pediatric obesity involves distinct developmental, psychosocial, and clinical considerations that fall outside the scope of this review.

The context included all healthcare educational settings in which obesity suits were employed to foster learning. This included activities focusing on empathy, communication skills, stigma awareness, clinical skills, or broader professional development within undergraduate or postgraduate curricula. To be considered applicable, an educational activity had to incorporate some form of evaluation. Studies were excluded if obesity suits were used in non-educational settings such as entertainment, theatre, or costume design, or in training contexts unrelated to healthcare education.

To provide a comprehensive overview, a wide range of study designs was included, encompassing quantitative, qualitative, and mixed-methods studies, as well as project reports and grey literature such as theses and conference abstracts.

### Data selection and extraction

**Fig. 1:**
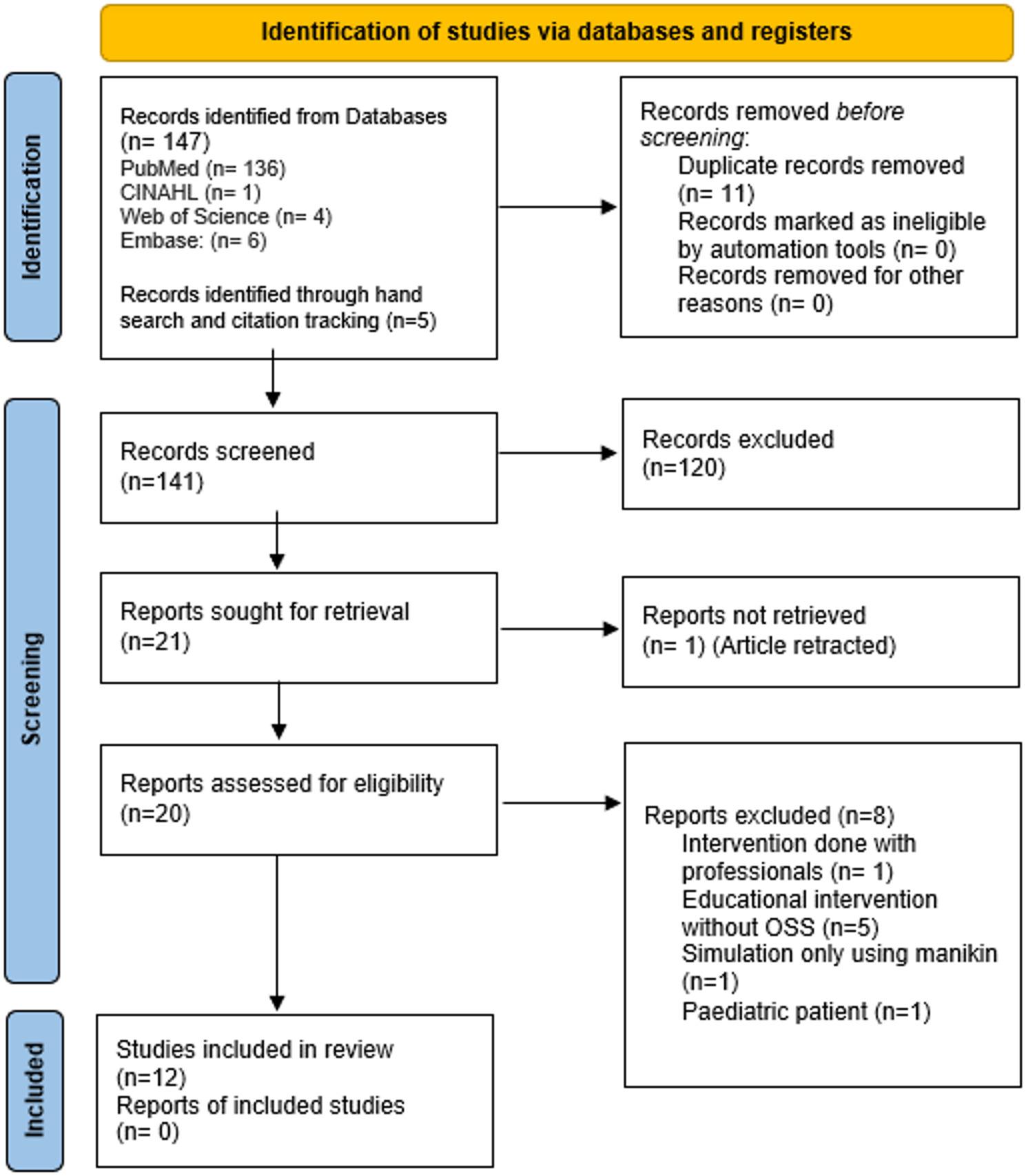
PRISMA flow chart diagram

The database search yielded 147 records; an additional five studies were identified through citation tracking. The study selection process is presented in the PRISMA flow diagram (Fig. 1).

The first author (AS) conducted the search. The screening of titles and abstracts and full-text data extraction was performed independently by AS and LB. Extracted datasets were then compared, and any discrepancies were resolved through discussion; when needed, VK and DH served as a third reviewer.

### Data charting, analysis and synthesis

After the independent extraction phase, the final dataset was structured using MAXQDA (Verbi GmbH, Berlin, Germany) [22]. A standardized data charting form was used to extract and organize key study characteristics, including bibliographic information (author, year, country), participant details, type of intervention, and reported outcomes. The form was developed based on the review protocol and was piloted on two studies to ensure consistency and clarity.

For this purpose, a data extraction tool was developed by the research team in accordance with the recommendations from Pollock et al. [23]. Data synthesis followed a descriptive and thematic approach guided by the four research questions. Data were categorized deductively according to these questions, which served as an analytical framework for organizing and presenting the findings.

Consistent with guidance for scoping reviews, no formal critical appraisal of included studies was conducted. Given the limited number of available studies and the substantial heterogeneity in study designs, educational contexts, and outcome measures, a formal quality assessment was considered unlikely to meaningfully support the synthesis.

## Results

The database search identified 147 records, and five additional studies were retrieved through citation tracking. Following removal of duplicates, titles and abstracts were screened, and full texts of potentially relevant articles were assessed for eligibility. Twelve studies met the inclusion criteria and were included in the review [14, 16, 17, 24–32]. The full selection process is presented in Fig. 1.

Seven studies employed quantitative designs [14, 16, 24–27, 30], two used qualitative methodologies [17, 29], and two applied mixed-methods approaches. One study was a descriptive project report without a clearly defined systematic methodology [31].

Across the included studies, most focused on participants’ subjective experiences and reactions to wearing OSS, typically assessed through self-reported measures or reflective accounts. In addition, two studies incorporated objective physiological measures [17, 28], such as heart rate and blood pressure.

Most studies (*n* = 6) originated in the USA [17, 26, 28–31], two in Germany [14, 27], and one each in Israel [16], Switzerland [24], Canada [32] and Türkiye [25]. An overview of the included studies is presented in Table 1.

**Table 1:** Table of included studies

Title of Study	Authors, Year, Country	Participants	Use of the Obesity Simulation Suit	Results
Examining the effect of an obesity suit role-playing exercise on empathy and weight bias in nutrition students	Tayar Wachsberger et al., 2025, Israel [16]	34 Nutrition Science Students	At Ariel University’s Simulation Center, students wore an OSS (+ 18 kg) during a 15-minute standardized clinical scenario with an actor portraying a dietitian. The scenario incorporated weight-biased elements (e.g., inappropriate equipment). Validated through feedback from dietitians and individuals with obesity. Pre/post surveys assessed participants’ perceptions.	The intervention used the OSS paired with a simulation scenario led to a reduction in implicit weight bias scores among nutrition science students. No significant changes in general anti-fat attitudes, fat-phobia, or levels of empathy were reported
Exploring physiological and emotional responses to exercise with additional body mass: an experiential learning activity	Ruegsegger, 2025, USA [28]	12 EXSS undergraduates	The Exercise for Special Populations students completed treadmill walking and cycling with and without a 15% weighted vest to simulate additional body mass, following a preparatory lecture on exercise testing and assessment; physiological and psychological responses were monitored, and post-activity feedback and reflections assessed learning and empathy development	Students experienced heightened physiological exertion and emotional challenge while exercising with added mass, finding the actual activity more demanding than anticipated. Feedback following the exercise revealed increased confidence in exercise testing and a deeper awareness of the difficulties encountered by individuals with overweight, which supports improved empathy and practical skills.
Fostering equity in nursing education and practice: Inclusive simulation for weight bias reduction	Marsack et al., 2025, USA [30]	130 Nursing Students	Sophomore Nursing students participated in an educational intervention focused on the care of overweight and obese patients. The intervention included the use of obesity suits during simulated patient care activities, aimed at addressing weight bias. Pre- and immediate-post surveys were conducted to evaluate their attitudes, beliefs, and cultural humility towards individuals who are overweight or obese.	This educational intervention positively influenced undergraduate nursing students’ perceptions of patients with obesity, potentially fostering more inclusive practice skills, diminishing weight biases, and enhancing the quality of patient care.
Mindfulness‑based empathy training supported by Obese Simulation Suit: Randomized Controlled Trial	Can Gür & Yilmaz, 2024, Türkiye [25]	84 Nursing Students	The Mindfulness-Based Empathy Training with the OSS was a 6-week program (12 sessions, 40–50 min each) combining mindfulness practices with empathy-focused activities. Two sessions included expert-led education on obesity, while the remaining sessions were delivered by the researcher. The OSS was integrated into the training to enhance perspective-taking toward individuals with obesity	Nursing students in the experimental group showed significant improvements in both attitudes and empathy toward individuals with obesity compared to controls. Gains were sustained across pretest, posttest, and follow-up measures, highlighting the effectiveness of OSS in fostering positive competencies for patient care
An Undergraduate Baccalaureate Simulation on Weight Bias and Caring for a Patient with Obesity	Andrews, 2024, USA [29]	112 nursing students	A pilot learning module for BSN students combined a simulation with a standardized patient wearing a bariatric suit, followed by a lecture on weight bias, evidence-based strategies for patient care, and reflective exercises; learning and patient experience were evaluated through reflections and SP surveys	Most students identified weight bias (91%) and suggested interventions to enhance patient experience (71%) and outcomes (85%), highlighting the effectiveness of simulation as a tool for teaching compassionate, patient-centered care.
Fostering weight status understanding among exercise science and health students by simulating common physical activities with additional body mass	Ladwig, 2023, USA [17]	9 Exercise Science and Health Students	The Exercise Science and Health students engaged in a two-part activity comprising a didactic lesson on the psychophysiological responses to physical activity, followed by simulations of four tasks—shoe tying, walking, running, and climbing stairs—while wearing weighted vests of 16 pounds and 32 pounds to simulate additional body mass. The students predicted outcomes, performed the tasks, and reflected on their experiences, as well as strategies to encourage physical activity among overweight clients.	Students’ reflections demonstrated a heightened awareness of the psychophysiological challenges associated with physical activity when carrying additional weight, especially following the 32-pound simulations. This suggests an increase in empathy and a potential reduction in weight bias.
Effects of weight bias training on student nurse empathy: A quasi-experimental study	Gajewski, 2023, USA [26]	121 Nursing Students	In a university simulation lab, an SP wore an OSS during a hospital room scenario. Nursing students interacted with the SP, who followed a structured script with cues about physical barriers related to weight (e.g., too-small chair). The SP was trained to ensure fidelity and realism; the scenario was piloted prior to the study.	Overall empathy scores showed no significant pre/post change. However, the accelerated second-degree cohort demonstrated a small but significant improvement, while the traditional cohort did not. Patient-rated empathy was mostly moderate, and student comments indicated persistence of weight bias.
The Effect of a Multifaceted Intervention Including Classroom Education and Bariatric Weight Suit Use on Medical Students’ Attitudes toward Patients with Obesity	Renold et al., 2023, Switzerland [24]	79 Medical students	As part of an 8-session semester course on obesity, students participated in a gamification task using a 7 kg OSS. In groups, two students wore the suit for ≥ 30 min in public settings (e.g., transport, shopping, restaurants) while one observed social reactions. Experiences were documented and later presented and discussed in class	Overall attitudes (NEW Attitudes Scale) did not change significantly pre- vs. post-intervention, though 4th-year medical students showed improvement (*p* = .02). Nine survey items shifted significantly, including reduced agreement with stereotypes such as ‘individuals with obesity lack willpower’. Effects were limited in students with low baseline bias
Improving obesity management training in family medicine: multi-methods evaluation of the 5A*sT-MD pilot course	Luig et al., Canada, 2020 [32]	42 Family Medicine Residents	The course integrates lectures with practical experiences, including wearing an OSS that simulates obesity, allowing residents to perform daily tasks in a Smart Condo. This hands-on experience is followed by narrative reflections and group discussions to deepen understanding and empathy. Residents then apply their skills in standardized patient interviews and in-clinic practice, using the 5 A’s framework for obesity management	Following the course, residents reported enhanced attitudes and confidence in counseling patients with obesity despite no notable change in ATOP scores. They demonstrated progress in assessing and advising on weight gain causes and treatment options, achieving health outcomes, and counseling on various related issues, including pregnancy, mental health, and referrals (all *p* < .05 to *p* < .01). Qualitative analyses of narrative reflections highlighted the importance of experiential learning in developing empathy and critical reflective skills.
Is an obesity simulation suit in an undergraduate medical communication class a valuable teaching tool? A cross-sectional proof of concept study	Hermann-Werner et al., 2019, Germany [14]	207 Medical Students	Within the basic communication curriculum, students participated in a SP encounter using the OSS. In small groups, one student acted as the physician interviewing an SP with type 2 diabetes, focusing on lifestyle habits and psychosocial factors. Sessions were moderated by experienced teachers, followed by feedback and questionnaires from students, teachers, and SPs	The OSS enhanced realism of the patient encounter according to students, teachers, and SPs, with BMI estimates consistently in the obese range. While overall stigma was low, students showed higher anti-fat attitudes than teachers or SPs. The OSS was considered a valuable tool for raising awareness
Integrating an Obesity Simulation into Baccalaureate Nursing Education	Mangold & Markiewicz, 2014, USA [31]	20 Nursing Students	In this simulation, students interacted with an SP dressed to simulate obesity, which was aimed at increasing realism. This setup helped students reduce anxiety and improve their interpersonal skills when engaging with unfamiliar individuals. It also gave them the opportunity to establish caring relationships and practice empathetic and compassionate nursing care for sensitive health issues.	In the simulation, students participated in an evolving case study within a baccalaureate nursing course, allowing them to provide care in a safe environment. This experience enhanced their problem-solving skills and deepened their understanding of the chronic nature of obesity and its associated challenges.
Instant Adipositas– Übergewicht selbst erleben	Werner et al., 2011, Germany [27]	Medical students and nutrition science students 135	Students wore an OSS and completed six tasks simulating daily challenges faced by people with obesity. The challenges were: tying shoes, sitting in a small chair, getting up from the ground, Walking upstairs, sprinting for 25 m and getting into a small car and fasten the seatbelt.	Post-simulation, reported empathy and understanding of psychological problems in people with obesity increased substantially

*5As of Obesity Management = ASK, ASSESS, ADVISE, AGREE, ASSIST

*Abbreviations*: *BMI* Body Mass Index, *OSS* Obesity Simulation Suit, *EXSS* Exercise Science Students, *ATOP* Attitudes Toward Obese Persons, *SP* Standardized Patient, *BSN* Bachelor of Science in Nursing

### RQ1: Who are the target populations for OSS in healthcare education, and at what stage of training (undergraduate, postgraduate, or continuing professional development) are they employed?

OSS were implemented with learners from a range of health-related professions. Participants included medical students [14, 24, 27, 32], nutrition science students [16, 27], nursing students [25, 26, 29–31], and exercise science students [17, 28]. One study included both nutrition and medical students in the sample [27].

Several studies implemented OSS during the early to mid undergraduate phase. These included sophomore-level students [14, 30] and first-semester undergraduate students [16, 26]. Other studies integrated OSS into advanced undergraduate or senior-level training, including senior-year students [29], senior-level courses [28]. One study implemented OSS in postgraduate training [32].

### RQ2: In what ways are OSS incorporated into educational activities, and what additional teaching or learning methods are combined with their use?

In the included studies, OSS-based activities were implemented with a variety of other educational formats that typically combined prebriefing, experiential tasks, and structured reflection. In several cases, OSS use formed one component within a broader, multi-session or semester-long curriculum on obesity, communication, or patient-centered care [24, 25, 32].

Most studies implemented a prebriefing before learners engaged with OSS. These included didactic lectures covering weight bias, obesity etiology, communication principles, psychosocial factors, and clinical management considerations [28, 29, 32]. Preparations also included assigned articles on weight bias or professional guidelines [26], and videos, such as TED talks or narrative patient accounts describing experiences of stigma or healthcare interactions [24, 26]. Structured prebriefings were commonly implemented to orient students to learning objectives, roles, procedures, and available equipment, designed to ensure psychological safety and clarity before entering the simulation [14, 29].

Two primary modes of interaction with OSS were identified: (1) learners wore the OSS themselves to experience simulated physical and functional constraints, and (2) the OSS was worn by a standardized or simulated patient (SP), with students assuming the role of caregivers during clinical scenarios. A core component of many interventions involved learners physically performing tasks while wearing OSS or weighted vest to simulate increased body mass. Reported activities included: Activities of daily living such as shoe tying, getting dressed, bending, or cleaning [27, 32], mobility-related tasks, including walking, running, climbing stairs, transfers, rising from the floor, and entering or exiting vehicles [17, 27, 30] and exercise-based protocols, such as treadmill or cycling tasks performed with and without added body mass [28]. Some studies required learners to alternate roles, acting both as a caregiver and as an SP wearing the suit [30].

Several interventions used SP wearing OSS to simulate clinical encounters with individuals living with obesity. These scenarios included conducting focused health assessments in a simulated hospital room, with cues embedded to highlight environmental barriers [26] or performing nutrition or lifestyle counselling, informed by intentionally varied levels of weight bias presented by the SP or scenario design [16]. Another intervention was medical history taking and exploration of psychosocial contributors to chronic conditions such as diabetes [14]. These scenarios emphasized communication, empathy, and awareness of equipment or environmental constraints relevant to individuals living with obesity.

All studies included post-simulation debriefing or reflection. Debriefings commonly encouraged learners to reflect on their own assumptions, emotional responses, and communication strategies; to discuss observed weight bias; and to identify improvements for patient-centred care [29, 30]. Some studies incorporated written reflections or narrative assignments, which were later reviewed in small-group discussions facilitated by instructors or expert preceptors [32].

### RQ3: How do learners respond to the experience of using OSS, in terms of their reported perceptions, attitudes, and learning outcomes?

Across included studies, a range of validated questionnaires was used to assess changes in attitudes, beliefs, empathy, and implicit bias. Several measures were used in more than one study, allowing for comparison across interventions. The Attitudes Toward Obese Persons (ATOP) scale was used in three studies [25, 30, 32]. One study reported a significant improvement in ATOP scores from pre- to post-intervention [30]. A second reported no significant change following the intervention [32]. A third found a significant increase in attitudes in the experimental group compared to the control group, with significant changes across pretest, posttest, and follow-up assessments [25]. The Beliefs About Obese Persons (BAOP) scale was used in two studies. One study reported stable scores pre- to post-intervention [30], while the other reported significant improvement [32]. The Implicit Association Test (IAT) for weight bias was used in one study which reported significant reductions in implicit bias following the intervention [16]. The Cultural Humility Scale (CHS) was used in one study, which reported high baseline scores that remained stable post-intervention, with non-significant increases in half of the items [30]. The Fat Phobia Scale was used in one study, which reported a modest but non-significant reduction in fat-phobia scores, with mean scores remaining within the negative-attitude range throughout the study period [16]. The NEW Attitudes Scale was used in one longitudinal educational program. Pre- and post-intervention scores remained stable over time and between student cohorts [24]. Two studies assessed empathy quantitatively. Using the Jefferson Empathy Scale for Nursing Students, one study reported significant improvements in empathy scores in the intervention group compared to controls [25]. Another reported no significant changes [16].

Qualitative data from reflective narratives, open-ended survey responses, and written assignments described a range of physical, emotional, and cognitive responses to interventions. Participants reported physical sensations such as breathlessness, exhaustion, fear of falling, and efforts to avoid unnecessary energy expenditure while wearing OSS [17, 32]. Emotional responses included shock, shame, embarrassment, and self-consciousness when imagining themselves in a larger body [32]. Reflections indicated increased awareness of barriers related to physical activity while carrying additional mass, particularly in simulations involving higher weighted loads [17]. Learners also described experiences of increased self-reflection and empathic engagement during and after the intervention activities [32]. Some studies reported anecdotal positive outcomes noted by both learners and faculty related to the simulation activities [31]. Simulation experiences embedded within broader nursing education courses were reported to support understanding of the chronic and multidimensional aspects of obesity and to facilitate problem-solving in a supported learning environment [31].

### RQ4. What limitations or concerns related to the use of OSS in healthcare education are reported in the literature?

Three studies identified limitations in using OSS to represent lived experiences of individuals with obesity [16, 25, 28]. Ruegsegger noted that chronic joint stress, altered gait, metabolic factors, and especially psychological experiences such as stigma and long-term self-perception cannot be fully reproduced in short-term simulations [28]. The study suggested that including individuals with lived experience of obesity in future learning activities could offer additional insight into these psychological and social dimensions. Tayar-Wachsberger et al. similarly emphasized that OSS cannot fully replicate the lived experiences of people with obesity [16]. Can Gür and Yilmaz reported that the brief duration of suit use does not adequately capture the complex, multifaceted realities of living with obesity [25]. In addition to these criticisms, one study reported learner feedback that reflected similar concerns: two participants expressed uncertainty about the purpose of the exercise and perceived the simulation as ineffective for understanding obesity-related experiences [32].

## Discussion

This scoping review identified 12 studies examining the use of OSS in health professions education. Across these studies, OSS were implemented in two main ways: either learners wore the suits themselves [17, 24, 25, 27–30, 32] or they interacted with SPs wearing the suit [14, 16, 26, 31]. In most cases, OSS were embedded within broader educational interventions, including lectures, reflective exercises, or curricular activities.

The findings of this review indicate that the effects of OSS interventions are mixed and generally modest. Some studies reported improvements in attitudes, beliefs, empathy, or implicit bias [25, 30], while others found no significant changes [16, 32]. Differences were also evident across commonly used instruments such as the Attitudes Toward Obese Persons (ATOP), Beliefs About Obese Persons (BAOP), and Implicit Association Test (IAT), suggesting that OSS may influence certain dimensions of attitudes without consistently affecting others [16, 25, 30, 32].

Importantly, the included studies relied almost exclusively on self-reported outcomes assessed immediately after the intervention, and no study reported long-term follow-up. As a result, it remains unclear whether observed changes reflect sustained shifts in attitudes or merely short-term reactions to the simulation experience. Qualitative findings further support this interpretation: while learners frequently described strong physical and emotional responses [17, 32], these experiences did not consistently translate into measurable or lasting changes in bias or empathy. In addition, several studies implemented OSS as part of multicomponent interventions [32], making it difficult to isolate the specific contribution of the simulation suit to the reported outcomes. Collectively, the findings suggest that OSS may be effective in prompting reflection and increasing awareness, but current evidence does not demonstrate consistent or durable effects on weight bias or empathy.

Beyond effectiveness, this review also examined how included studies addressed limitations of OSS. Three studies explicitly acknowledged that OSS cannot authentically reproduce the lived experience of individuals with obesity [16, 25, 28]. These studies highlighted that aspects such as long-term adaptation, psychological burden, and the pervasive impact of weight stigma cannot be captured in short-term simulation exercises.

These observations are consistent with critiques in the broader literature. Authors [18, 33] argue that OSS risk reducing obesity to physical constraints, thereby neglecting the complex psychological, social, and temporal dimensions of the condition. Similarly, the temporary and reversible nature of wearing a simulation suit contrasts with the chronic and evolving experience described by individuals living with obesity. Experiences such as internalized stigma and long-term social marginalization are difficult to replicate within controlled educational settings.

A further concern is that perspective-taking exercises may not uniformly reduce stereotypes. As suggested by Skorinko and Sinclair [34], learners may interpret simulated experiences through pre-existing assumptions, potentially reinforcing rather than challenging bias. This is supported by Nario-Redmond et al. [35], who caution that patient-perspective exercises involving stigmatized conditions can, under certain circumstances, strengthen negative perceptions rather than mitigate them.

Taken together, these findings suggest that OSS should be used with careful pedagogical consideration. While OSS offer practical advantages, as they are relatively low-cost, easy to implement, and can provide an embodied starting point for reflection, they appear insufficient as standalone interventions for addressing weight stigma.

The reviewed studies and broader literature indicate that the educational value of OSS may depend heavily on how they are integrated into teaching. In particular, structured pre-briefing, guided reflection, and debriefing appear essential to contextualize the experience and prevent misinterpretation [31]. Debriefing, in particular, is widely regarded as a core component of simulation-based and experiential learning and should be systematically integrated following such activities [36, 37].

Embedding OSS within broader, multimodal educational approaches, including active learning components, may help address some of their limitations. Evidence from obesity education research more generally suggests that such multimodal approaches, particularly those integrating interactive and experiential formats, can be more effective in addressing complex topics such as weight stigma and fostering meaningful attitudinal change [13, 38, 39]. To maximize their educational value, OSS should therefore be integrated from the outset into well-structured teaching designs rather than used as isolated interventions [2].

Several studies and external sources point to complementary or alternative strategies. Encounters with SPs living with obesity have been shown to improve empathy and communication skills [40], while also providing more authentic insights into lived experiences. However, such approaches require careful preparation and may place additional demands on participants with lived experience. Virtual reality (VR)–based simulations have also been proposed as an alternative, offering standardized and controlled learning environments [41]. While promising, evidence regarding their effectiveness in reducing weight bias remains limited. Rather than replacing OSS, these approaches may be best understood as complementary. Combining OSS with patient narratives, SP encounters, or other reflective methods may provide a more comprehensive and ethically sensitive approach to obesity education.

### Strengths and limitations

This scoping review provides, to our knowledge, the first systematic mapping of how OSS are used across health professions education. By synthesizing evidence from a range of professional fields, including medicine, nursing, nutrition, physiotherapy, and other allied health disciplines, the review offers an overview of diverse educational applications. A further strength is the inclusion of both peer-reviewed and grey literature, which enabled the identification of emerging practices that may not yet be represented in indexed databases. The inclusion of studies from several countries also reflects the growing international interest in simulation-based approaches to obesity education.

Several limitations should be considered. As is characteristic of scoping reviews, the aim was to map the breadth of available evidence rather than to formally assess study quality; therefore, conclusions regarding effectiveness should be interpreted with caution. The number of included studies was limited, and interventions varied considerably in design, duration, learner population, and outcome measures. This heterogeneity restricted comparability across studies and contributed to the mixed findings observed, particularly with regard to attitudes and empathy. In addition, OSS were frequently embedded within multicomponent educational interventions, making it difficult to determine the specific contribution of the simulation suit to reported outcomes. Finally, most studies relied on self-reported measures assessed immediately after the intervention, with little or no long-term follow-up, limiting insights into sustained learning or behavioural change.

## Conclusion

The findings of this review suggest that OSS represent one of several approaches used in health professions education to address weight stigma and promote perspective-taking. Across the included studies, OSS were associated with increased awareness of physical challenges related to higher body weight and, in some cases, short-term improvements in selected outcomes such as attitudes or implicit bias. However, results were inconsistent, and not all studies reported significant changes.

At the same time, both the included studies and the broader literature highlight important limitations. OSS primarily simulate selected physical aspects associated with higher body weight and may not capture the complex psychological, social, and long-term dimensions of lived experience. This limitation was also reflected in the reviewed studies, several of which explicitly noted that simulation-based experiences cannot fully reproduce the realities of living with obesity.

Taken together, these findings indicate that OSS may serve as a useful introductory or complementary tool within broader educational strategies, rather than as a standalone intervention. Approaches that combine OSS with reflective discussion, patient narratives, or lived-experience perspectives may help to address some of the identified limitations and support more comprehensive learning.

Further research is needed to better understand how different educational strategies contribute to reducing weight stigma in healthcare. In particular, future studies should explore multimodal approaches, include longer-term follow-up, and examine how the integration of lived experience can support more nuanced and stigma-sensitive education.

## Supplementary Information

Additional file 1.

Additional file 2.

## Data Availability

The datasets generated during the current study are available from the corresponding author on reasonable request.
