# Supplementary material for: Obesity simulation suits in health professions education: a scoping review
Source: BMC Med Educ. 2026 Jun 2;26:894. doi: 10.1186/s12909-026-09565-1 (PMC13231796; doi:10.1186/s12909-026-09565-1)
Supplement: Supplementary file 1 — Additional file 1. [file 12909_2026_9565_MOESM1_ESM.pdf]

Additional File 1

Obesity Simulation Suits in Health Professional Education: A Scoping Review

Authors

Steinacker, Anna Christine; Klingenberg, Michael; Bischof, Lukas, Herchet, Daniela; Kreiss, Victoria, Bösner Stefan

Day of Search 08.11.2025

Pubmed Search Strategy

#1	**"medical education" OR "nursing education" OR "healthcare education" OR "Students, Medical"[Mesh] OR "Students, Nursing"[Mesh] OR "Students, Health Occupations"[Mesh] OR "Nursing student*" OR "Medical student*" or "Nutrition Student*" OR" Health Student*" OR "Physical Therapy Student*" OR "physiotherapy student***	463,764
#2	**“obesity suit" OR "obesity simulation suit" OR "fat suit" OR "bariatric suit" OR "Obese suit" OR "obese simulation suit" OR „body-weight simulation" OR „obesity simulation experience" OR „body size simulation" OR „weighted vest simulation" OR „obesity empathy intervention" OR „fatness simulation" OR „bariatric patient simulation" OR „obesity role-play"**	15,320
	#1 AND #2	136

CINAHL Search Strategy

#1	**"Nursing student*" OR "Medical student*" or "Nutrition Student*" OR" Health Student*" OR "Physical Therapy Student*" OR "physiotherapy student*" OR “medical education" OR "nursing Education" OR "healthcare education"**	156,125
#2	**“obesity suit" OR "obesity simulation suit" OR "fat suit" OR "bariatric suit" OR "Obese suit" OR "obese simulation suit" OR „body-weight simulation" OR „obesity simulation experience" OR „body size simulation" OR „weighted vest**	15

	**simulation" OR „obesity empathy intervention" OR „fatness simulation" OR „bariatric patient simulation" OR „obesity role-play"**	
	#1 AND #2	1

Web of Science Search Strategy

#1	**""Nursing student*" OR "Medical student*" or "Nutrition Student*" OR" Health Student*" OR "Physical Therapy Student*" OR "physiotherapy student*" OR "medical education" OR "nursing Education" OR "healthcare education"**	162,453
#2	**"obesity suit" OR "obesity simulation suit" OR "fat suit" OR "bariatric suit" OR "Obese suit" OR "obese simulation suit" OR „body-weight simulation" OR „obesity simulation experience" OR „body size simulation" OR „weighted vest simulation" OR „obesity empathy intervention" OR „fatness simulation" OR „bariatric patient simulation" OR „obesity role-play"**	15
	#1 AND #2	4

Embase Search Strategy

#1	**'nursing student'/exp OR 'nursing student' OR 'allied health student'/exp OR 'allied health student' OR 'dietetics student'/exp OR 'dietetics student' OR 'health student'/exp OR 'health student' OR 'medical student'/exp OR 'medical student' OR 'physical therapy student'/exp OR 'physical therapy student' OR 'nursing student*' OR 'medical student*' OR 'physical therapy student*' OR 'nutrition student*' OR 'medical education'/exp OR 'medical education' OR 'nursing education'/exp OR 'nursing education' OR 'healthcare education''**	**759,070**
#2	**'obesity suit' OR 'obesity simulation suit' OR 'fat suit' OR 'bariatric suit' OR 'obese suit' OR 'obese simulation suit' OR 'body-weight simulation' OR 'obesity simulation experience' OR 'body size simulation' OR 'weighted vest simulation' OR 'obesity empathy intervention' OR 'fatness simulation' OR 'bariatric patient simulation' OR 'obesity role-play'**	**19**
	**#1 AND #2**	**6**
